# Do healthcare providers consider the social determinants of health? Results from a nationwide cross-sectional study in the United States

**DOI:** 10.1186/s12913-024-10656-2

**Published:** 2024-03-04

**Authors:** Jeffrey Glenn, Gwen Kleinhenz, Jenna M.S. Smith, Robert A. Chaney, Victor B.A. Moxley, Paola G. Donoso Naranjo, Sarah Stone, Carl L. Hanson, Alisha H. Redelfs, M. Lelinneth B. Novilla

**Affiliations:** 1 Department of Public Health, Brigham Young University, 84602 Provo, UT USA; 2 J. Reuben Clark Law School, Brigham Young University, 84602 Provo, UT USA

**Keywords:** Social determinants of health, Social needs, Healthcare providers

## Abstract

**Background:**

While the social determinants of health (SDOH) have a greater impact on individual health outcomes than the healthcare services a person receives, healthcare providers face barriers to addressing these factors in clinical settings. Previous studies have shown that providers often lack the necessary knowledge and resources to adequately screen for and otherwise assist patients with unmet social needs. This study explores the perceptions and behaviors related to SDOH among healthcare providers in the United States (US).

**Methods:**

This cross-sectional study analyzed data from a 22-item online survey using Reaction Data’s research platform of healthcare professionals in the US. Survey items included demographic questions as well as Likert scale questions about healthcare providers’ perceptions and behaviors related to SDOH. Descriptive statistics were calculated, and further analyses were conducted using t-tests and analysis of variance.

**Results:**

A total of 563 respondents completed the survey, with the majority being male (72.6%), White (81%), and located in urban areas (82.2%). In terms of perceptions, most providers agreed or strongly agreed that SDOH affect the health outcomes of all patients (68.5%), while only 24.1% agreed or strongly agreed that their healthcare setting was set up to address SDOH. In terms of behavior, fewer than half currently screened for SDOH (48.6%) or addressed (42.7%) SDOH in other ways. Most providers (55.7%) wanted additional resources to focus on SDOH. Statistical analyses showed significant differences by gender, with females being more likely than males to prioritize SDOH, and by specialty, with psychiatrists, pediatricians, and family/general medicine practitioners being more likely to prioritize SDOH.

**Conclusion:**

Most healthcare providers understand the connection between unmet social needs and their patients’ health, but they also feel limited in their ability to address these issues. Ongoing efforts to improve medical education and shift the healthcare system to allow for payment and delivery of more holistic care that considers SDOH will likely provide new opportunities for healthcare providers. In addition to what they can do at the institutional and patient levels, providers have the potential to advocate for policy and system changes at the societal level that can better address the root causes of social issues.

**Supplementary Information:**

The online version contains supplementary material available at 10.1186/s12913-024-10656-2.

## Background

The social determinants of health (SDOH)—which the World Health Organization defines as “the conditions in which people are born, grow, work, live, and age, and the wider set of forces and systems shaping the conditions of daily life”—affect a wide range of health outcomes [1]. A large body of evidence suggests that SDOH likely have a more significant impact on an individual’s health than the healthcare services a person receives [2–4]. In addition, research has revealed the impact of SDOH on in-utero infant development [5], youth development [6, 7] and adult health [8]. Because they regularly interact closely with large numbers of patients, healthcare providers (i.e., people or places—such as doctors, nurses, or hospitals—that are licensed to give health care) [9] have the potential to play a critical role in reducing health disparities by addressing patients’ social needs (e.g., housing, food, financial support, and other social factors) [10, 11], yet a variety of barriers inhibit providers from dedicating time and financial resources towards these efforts [12, 13].

Frameworks for addressing SDOH from within the healthcare sector suggest that healthcare providers can take action at the community/societal level, practice/institutional level, and patient level [12, 14]. At the community level, providers are well-positioned to support their patients and advocate for policy and system-level changes that address the upstream social causes of health disparities [12]. Within healthcare institutions and at the patient level, interventions to address patients’ social needs range from passive data collection and referral services to direct interventions such as providing on-site services like legal advice and food pantries [13]. Of course, potential interventions at the institutional and patient levels depend on the characteristics of each specific determinant, as some social needs are more amenable to intervention by providers (e.g., food insecurity) than other social needs (e.g., financial precarity) [12, 13]. Key elements that lead to successful approaches are an appropriately staffed and trained workforce, health information technology innovations, and new financing models, such as those being tested within some state Medicaid programs, that are designed to incentivize value-based care rather than merely volume of services [13, 15].

One of the primary ways healthcare providers are expanding beyond their traditional medical service delivery role to address SDOH is by screening patients for social concerns and referring them to community services that can address identified needs [12, 16, 17]. Organizations such as the National Academy of Medicine (NAM), the American Academy of Pediatrics (AAP), and the American Academy of Family Physicians (AAFP) now recommend screening for SDOH risk factors during patient encounters [13, 18, 19]. Multiple evidence-based screening models exist to assist healthcare providers in screening for SDOH needs [20, 21]. For example, the WE CARE model (Well Child Care, Evaluation, Community Resources, Advocacy, Referral, Education) is a commonly used approach that has been found to be effective [22]. The Center for Medicare and Medicaid Services (CMS) and the National Association of Community Health Centers (NACHC) have also created screening tools that have been tested and are available for widespread use [13].

Major barriers remain in implementing SDOH screening programs and initiatives within the healthcare sector. Some argue that since screening for SDOH is outside the scope of clinical care, it may be ineffective and even unethical to screen if there is no capacity to ensure linkages to appropriate services [20, 23]. Questions also remain on who should conduct screenings and what training and other experience should be required; the sensitive nature of asking about social issues may be uncomfortable for providers without the requisite capacity [20]. Without standardized guidelines, unintended consequences may result (e.g., failing to meet patients’ expectations) depending on who does the screenings and how prepared they are to refer to services [12, 20, 21]. Patients may be uncomfortable answering questions about their non-medical needs and may not want help addressing social needs even if they disclose them to their providers [24]. This work can also be time-consuming for providers who may feel overwhelmed and lack the time and resources to adequately screen for and address SDOH [12, 13, 25]. Furthermore, it has typically been difficult for providers to bill and receive payment for non-clinical services [13].

Provider perceptions about the importance of and strategies to address SDOH are essential to consider if we are to better address patients’ social needs in clinical settings, a practice that many healthcare organizations are currently attempting without sufficient foundational evidence [26]. While some researchers have attempted to answer these questions, previous studies have been limited in their scope with small samples and a focus only on specific health systems, geographic areas, or facilities. Thus, gaps remain in understanding provider perceptions, especially with large samples across specialties, facilities, and geographic areas [27, 28]. This exploratory study contributes to this discussion by exploring the perceptions and behaviors regarding SDOH among healthcare providers across different specialties and locations in the US.

## Methods

### Study design

This cross-sectional study sought to understand healthcare providers’ perceptions and behaviors related to SDOH. An online survey was administered in collaboration with Reaction Data, a healthcare market research firm that maintains a research database of more than 800,000 healthcare professionals who have consented to receive invitations from Reaction Data to participate in research [29]. Target respondents were US healthcare providers from a variety of specialties and healthcare settings, including ambulatory clinics, community hospitals, research hospitals, and integrated delivery networks (IDN).

### Survey instrument

A 20-item survey was developed by the research team based on a thorough review of relevant literature. Questions on perceptions and behavior were adapted from survey questions used in two previous studies [28, 30]. Reaction Data staff suggested revisions that were incorporated into the final instrument. The survey was administered in English and consisted of three demographic questions and 17 questions about providers’ perceptions of SDOH and current SDOH-related practices (see additional file “Final Survey instrument_SDOH Perceptions.pdf”). At the beginning of the questionnaire, respondents were provided with a basic definition of SDOH: “Social determinants of health (SDOH) are defined as the conditions in the environments where people are born, live, learn, work, play, worship, and age; such as housing, transportation, education, income, food access, neighborhood safety, water and air quality, employment, etc.” [31]. The demographic questions included gender, age, and race/ethnicity. In addition, Reaction Data was able to provide some demographic information (position, specialty, facility type, and zip code) on each respondent based on existing profiles found in the Reaction Data database. Participants were asked to use a 5-point numbered Likert scale (from “strongly disagree” to “strongly agree”) to respond to statements such as, “SDOH affects the health outcomes of all individuals within the US healthcare system” and “SDOH affects the health outcomes among patients in my healthcare setting.” Additionally, participants were asked to identify (using a 5-point numbered Likert scale from “all of the time” to “never,”) how often they engage in specific practices in their healthcare settings. These practices included thinking about SDOH, screening for SDOH, and addressing SDOH.

### Data collection

During March 2021, email invitations were distributed to physicians, nurses, and other healthcare workers on the Reaction Data platform. A cover letter contained information about the study and the link to the online survey. Each potential respondent received up to three email invitations to participate. Once the invitation link to the survey was clicked, no additional emails were sent. If the link was not clicked, up to two additional reminder emails were sent to the participants. All survey participants gave informed consent prior to participating and were eligible to withdraw from the survey at any point. No incentives were provided to participants in the study.

### Data analysis

Data were analyzed using R, version 4.2.2 [32]. Incomplete surveys were excluded from the analysis. Participant occupational specialties, employment facility, and geographic region were grouped into most similar categories for descriptive and inferential analysis, including group mean differences. Participant zip codes were aggregated regionally using census regions and categorically using Rural-Urban Commuting Area codes (RUCA), which classify areas based on population density, urbanization, and commuting activity [33, 34]. Independent t-tests and one-way analysis of variance were used to assess differences between groups.

## Results

A total of 563 healthcare workers responded out of 8,165 who received the survey, for a response rate of 6.9%. Among those (see Table 1) who responded, 409 were males (72.6%), and 141 were females (25%). The ages of participants ranged from 36 to 87 years, with 63.7% of the responses coming from participants in the 46–65-year-old age group. In terms of ethnicity, 81% were White and no other racial group constituted more than 8% of the sample. Of the 563 respondents, 463 (82.2%) were located in urban areas, predominantly from the Southern and Western regions of the US. Physicians (544) comprised 96.6% of the study population with 9 (1.6%) nurses; and 10 (1.8%) providers who worked in some other capacity. Most respondents were from Internal Medicine (19.5%), Surgery (14.9%), Anesthesiology (11.5%), Pediatrics (10.8%), and Family/General Medicine (8%) who worked in ambulatory clinics (38.7%), community hospitals (27.5%) and research hospitals (21%). The final sample of respondents may not be representative of the target population of healthcare providers in the US due to the sampling strategy used.

**Table 1:** Respondent demographic summary (*n* = 563)

	#	%
**Gender**		
Male	409	72.6
Female	141	25.0
No response	13	2.3
**Position**		
Physician	544	96.6
Nurse	9	1.6
Other	10	1.8
**Specialty**		
Internal Medicine	110	19.5
Surgery	84	14.9
Anesthesiology	65	11.5
Pediatrics	61	10.8
Family/General	45	8.0
Rehab/Chiropractic	38	6.7
Psychiatry	28	5.0
Other	132	23.4
**Facility Type**		
Ambulatory Clinic	218	38.7
Community Hospital	155	27.5
Research Hospital	118	21.0
Outpatient	44	7.8
Other	28	5.0
**Age**		
Over 75	33	5.9
66–75	84	14.9
56–65	150	26.6
46–55	127	22.6
Under 46	41	7.3
No response	128	22.7
**Race/Ethnicity**		
White	456	81.0
Multiracial	45	8.0
Hispanic	16	2.8
Black or African American	15	2.7
Asian	1	0.2
Other	6	1.1
No response	24	4.3
**Region**		
South	198	35.2
West	161	28.6
Midwest	112	19.9
Northeast	88	15.6
No response	4	0.7
**Urban/Rural**		
Urban core	463	82.2
Large town	29	5.2
Suburb of urban core	14	2.5
Rural	7	1.2
Small town	4	0.7
No response	46	8.2

### Overall

Mean responses (based on the 5-point Likert scale) and the percentage of respondents that agreed or strongly agreed (ASA) with each survey item are given in Table 2. For the survey items about provider perceptions, the highest mean (3.93) was for the statement, “SDOH affects the outcome of ALL individuals in the US healthcare system,” with 68.5% of respondents agreeing or strongly agreeing with this statement. The second highest mean (3.86) was for, “Collecting SDOH information would put an additional burden on health providers,” with 66.9% agreeing or strongly agreeing. The third highest mean was for, “SDOH affects the health outcomes of patients in ‘MY’ healthcare setting,” with 64.4% agreeing or strongly agreeing. The lowest mean (2.64) was for the statement, “At the present time my healthcare setting is set up to address the SDOH”, with only 24.1% agreement.

For the items pertaining to provider behaviors, the highest means were for the statements on “wishing for additional resources” (3.50) and “thinking about the impact of SDOH” (3.48), whereas the means for “screening for SDOH” (3.22) and “addressing SDOH in other ways” (3.08) were lower. Over 55% of providers said they wished for more resources or thought about the impact of SDOH some or all the time, compared to only 42.7% of providers who said they addressed SDOH in ways other than screening some or all the time. The lowest behavior item was for the statement, “I highly prioritize addressing SDOH in my healthcare setting,” with only 33.9% agreement.

**Table 2:** Average responses for SDOH survey questions of perceptions and behaviors

	M	SD	%ASA
**Perceptions** ^i^			
SDOH affects the health outcomes of ALL individuals in the US healthcare system	3.93	1.19	68.5
Collecting information on SDOH would put additional burden on providers in my healthcare setting	3.86	1.19	66.9
SDOH affects the health outcomes among patients in MY healthcare setting	3.84	1.09	64.4
Collecting information on SDOH will allow my healthcare setting to improve patient outcomes	3.71	1.23	60.1
I am confident in my understanding of SDOH	3.55	1.17	53
The benefits of collecting information on the SDOH outweigh the burden and risks	3.40	1.19	47.7
SDOH are more important to overall health than the healthcare individuals receive	3.12	1.21	39.3
At the present time my healthcare setting is set up to address the SDOH	2.64	1.14	24.1
**Behaviors**			
I highly prioritize addressing SDOH in my healthcare setting^i^	3.02	1.16	33.9
How often do you do the following in your healthcare setting?^ii^			
Wish for additional resources programs to address the SDOH of patients	3.50	1.26	55.7
Think about the impact of SDOH on patients	3.48	1.17	55.1
Screen for SDOH	3.22	1.31	48.6
Address the SDOH of patients in any way other than screening	3.08	1.27	42.7

%ASA = Percent that indicate Agree and strongly agree OR percent that indicate Some of the time and All of the time

i) Likert response options: Strongly agree-5, agree-4, neither-3, disagree-2, strongly disagree-1

ii) Likert response options: All of the time-5, some of the time-4, occasionally-3, rarely-2, never-1

### Gender differences

The overall mean scores for the Likert scale questions were higher among female than male respondents in 11 survey items where a statistical difference was observed (See Table 3). The responses to the statement, “I highly prioritize addressing SDOH in my healthcare setting” showed the largest difference between genders with an average of 3.42 among females and an average of 2.88 (*t*(248.23) *=* 4.85, *p* < 0.001) among males. For this statement, 51.4% of female providers agreed or strongly agreed compared to only 28% of male providers. The next largest difference was noted in the response to the statement, “The benefits of collecting information on the SDOH outweigh the burden and risks,” with 62.1% of females agreeing or strongly agreeing, compared to 42.8% of males. There was no detectable difference as to gender in their views of their healthcare setting being set up to address SDOH and that collecting SDOH information would allow their healthcare setting to improve patient outcomes.

**Table 3:** Gender differences in SDOH perceptions and behaviors

	Female			Male			Comparison		
	M	SD	%ASA	M	SD	%ASA	t	df	p
**Perceptions** ^i^									
SDOH affects the health outcomes of ALL individuals in the US healthcare system	4.24	1.12	79.7	3.82	1.2	65	3.69	254.4	0.001
Collecting information on SDOH would put additional burden on providers in my healthcare setting	4.04	1.2	74	3.79	1.19	64.2	2.12	237.09	0.041
SDOH affects the health outcomes among patients in MY healthcare setting	4.17	1.01	74.8	3.73	1.1	60.9	4.36	260.19	< 0.001
Collecting information on SDOH will allow my healthcare setting to improve patient outcomes	3.67	1.36	59.4	3.72	1.19	60	-0.44	214.61	0.662
I am confident in my understanding of SDOH	3.8	1.11	62	3.46	1.18	49.6	3.01	248.74	0.004
The benefits of collecting information on the SDOH outweigh the burden and risks	3.71	1.18	62.1	3.3	1.18	42.8	3.51	242.68	0.001
SDOH are more important to overall health than the healthcare individuals receive	3.39	1.16	49.3	3.04	1.21	36.3	3.06	251.99	0.004
At the present time my healthcare setting is set up to address the SDOH	2.71	1.16	24.6	2.6	1.14	23.2	0.94	233.95	0.379
**Behaviors**									
I highly prioritize addressing SDOH in my healthcare setting^i^	3.42	1.12	51.4	2.88	1.15	28	4.85	248.23	< 0.001
How often do you do the following in your healthcare setting?^ii^									
Wish for additional resources programs to address the SDOH of patients	3.84	1.17	65.7	3.38	1.29	53.5	3.83	253.51	< 0.001
Think about the impact of SDOH on patients	3.8	1.11	65.9	3.37	1.18	51.3	3.84	239.67	< 0.001
Screen for SDOH	3.51	1.27	59.7	3.13	1.32	45.2	3.02	238.79	0.004
Address the SDOH of patients in any way other than screening	3.41	1.27	54.1	2.96	1.25	38.2	3.59	225.21	< 0.001

%ASA = Percent that indicate Agree and strongly agree OR percent that indicate Some of the time and All of the time

i) Likert response options: Strongly agree-5, agree-4, neither-3, disagree-2, strongly disagree-1

ii) Likert response options: All of the time-5, some of the time-4, occasionally-3, rarely-2, never-1

### Specialty differences

The overall mean scores differed between provider specialties across nearly all SDOH perceptions and behaviors (See Table 4). Psychiatrists had the highest mean responses among all questions (3.89), followed by pediatricians (3.69) and family/general medicine practitioners (3.59); anesthesiologists had the lowest (3.07). The highest mean among all specialties was by psychiatrists to the statement, “SDOH affects the health outcomes among patients in MY healthcare setting” (4.50), with 92.8% agreeing or strongly agreeing. The single lowest mean among all specialties was by anesthesiologists to the statement, “At the present time my healthcare setting is set up to address the SDOH” (2.35), with only 9.2% agreeing or strongly agreeing.

**Table 4:** Specialty differences in SDOH perceptions and behaviors

	Anesthesiology			Family/Gen Med			Internal Medicine			Pediatrics			Psychiatry			Surgery			ANOVA	
	M	SD	%ASA	M	SD	%ASA	M	SD	%ASA	M	SD	%ASA	M	SD	%ASA	M	SD	%ASA	F	p
**Perceptions** ^**i**^																				
SDOH affects the health outcomes of ALL individuals in the US healthcare system	3.51	1.31	53.8	4.02	0.99	74.4	4.04	1.17	70.8	4.08	1.21	75	4.36	1.06	92.8	3.82	1.23	65.1	2.17	0.04
Collecting information on SDOH would put additional burden on providers in my healthcare setting	3.52	1.45	56.9	3.89	1.08	68.2	4.14	1.07	75.2	3.85	1.17	67.8	4	0.98	67.9	3.54	1.31	51.8	3.01	< 0.001
SDOH affects the health outcomes among patients in MY healthcare setting	3.54	1.23	52.3	3.98	1	68.1	3.88	1.07	66.7	4.18	0.96	77	4.5	0.64	92.8	3.81	1.18	62.6	3.89	< 0.001
Collecting information on SDOH will allow my healthcare setting to improve patient outcomes	3.72	1.18	61.6	3.4	1.14	44.2	3.75	1.12	62.1	3.6	1.4	58.4	3.79	1.03	57.1	4.05	1.15	72	1.93	0.06
I am confident in my understanding of SDOH	3.23	1.32	43	3.61	0.97	52.3	3.48	1.2	54.6	3.93	1.04	65	3.96	0.88	67.8	3.38	1.24	51.2	2.73	0.01
The benefits of collecting information on the SDOH outweigh the burden and risks	3.02	1.28	32.3	3.37	1.16	53.5	3.56	1.16	52.8	3.8	1.12	63.3	3.89	0.92	67.9	3.17	1.13	37.8	4.03	< 0.001
SDOH are more important to overall health than the healthcare individuals receive	3.11	1.26	40	3.41	1.21	52.3	3.11	1.26	40	3.25	1.19	41	3.68	1.02	57.1	2.83	1.14	25.7	2.4	0.02
At the present time my healthcare setting is set up to address the SDOH	2.35	1.02	9.2	2.88	1.14	34.9	2.5	1.14	18.3	3.16	1.16	42.6	2.82	1.09	25	2.49	1.21	23.2	3.52	< 0.001
**Behaviors**																				
I highly prioritize addressing SDOH in my healthcare setting^i^	2.65	1.23	20	3.23	1.15	41.9	3.03	1.2	34.5	3.3	1.05	42.6	3.68	0.77	64.3	2.89	1.04	23.5	3.22	< 0.001
How often do you do the following in your healthcare setting?^ii^																				
Wish for additional resources programs to address the SDOH of patients	3.1	1.32	46	3.8	1.1	68.3	3.77	1.25	65.4	3.69	1.18	64.4	4.04	1.11	80.8	3.31	1.21	50	3.83	< 0.001
Think about the impact of SDOH on patients	3.22	1.17	46	3.73	1.12	63.4	3.51	1.22	58.8	3.69	1.1	66.1	3.92	1.06	69.2	3.29	1.21	48.7	1.93	0.06
Screen for SDOH	2.54	1.31	20.6	3.59	1.12	61	3.32	1.31	52	3.73	1.11	71.2	4.12	0.91	80.8	3	1.39	42.3	7.13	< 0.001
Address the SDOH of patients in any way other than screening	2.38	1.28	20.6	3.54	1.23	53.6	3.17	1.22	44.2	3.66	1.17	64.4	3.77	1.31	69.2	2.82	1.23	34.2	7.66	< 0.001
*All questions*	3.07	1.26	38.64	3.57	1.11	56.62	3.48	1.18	53.49	3.69	1.14	61.45	3.89	0.98	68.67	3.26	1.21	45.24		

%ASA = Percent that indicate Agree and strongly agree OR percent that indicate Some of the time and All of the time

i) Likert response options: Strongly agree-5, agree-4, neither-3, disagree-2, strongly disagree-1

ii) Likert response options: All of the time-5, some of the time-4, occasionally-3, rarely-2, never-1

### Geographic location

Participant SDOH perceptions and behaviors were compared between geographic groupings. Census regions were used for broad in-country positioning, and no statistical differences were found between regions. RUCA classifications were used to characterize the nature of participant locations. Due to insufficient sample sizes in all RUCA categories, participant locations were grouped as urban or non-urban. Statistical differences by location type were found among five questions. Urban locations responded higher for “SDOH affects the health outcomes of ALL individuals in the US healthcare system” (t(59.74) = 5.44, *p* < 0.001, urban = 4.10, non-urban = 3.08) and “Wish for additional resources programs to address the SDOH of patients” (t(66.92) = 4.11, *p* < 0.001, urban = 3.63, non-urban = 2.92). There were several questions where participants from non-urban areas had higher responses: “Address the SDOH of patients in any way other than screening” (t(66.67) = 3.15, *p* = 0.002, urban = 3.00, non-urban = 3.57); “Screen for SDOH” (t(70.51) = 2.58, *p* = 0.01, urban = 3.19, non-urban = 3.62); “SDOH are more important to overall health than the healthcare individuals receive” (t(66.93) = 2.19, *p* = 0.03, urban = 3.06, non-urban = 3.42).

### Age differences

Age-based comparisons yielded few statistical differences. When grouping ages by 10-year increments, “SDOH are more important to overall health than the healthcare individuals receive” was the only statistically different question (F = 4.23, *p* < 0.001). Grouping birth years into two groups, 1934–1965 and 1965–1985, showed that older participants “Address the SDOH of patients in any way other than screening” less than younger ones (t(322.56) = 2.21, *p* = 0.02).

## Discussion

This study, comprised primarily of physician respondents from various healthcare facilities in the US, sought to better understand provider perceptions regarding SDOH across different specialties and locations. Findings showed that 68.5% of providers believe SDOH affect their patients’ health outcomes, but most feel their healthcare practices are not set up to address SDOH adequately. Overall, these findings echo the results obtained by previous studies. In a cross-sectional study of 240 clinical faculty in a university health system, Palacio et al. (2018) found that 83% of respondents agreed that SDOH were important predictors of health outcomes [28]. Likewise, in a study of 258 respondents from 14 medical centers, Schickedanz et al. (2019) found that 82% of physicians, nurses, case managers, pharmacists, and social workers agreed that social needs were an issue for most patients [25]. In a smaller study comprised of 43 residents, faculty, and staff at a pediatric health center, 100% of residents and 85% of faculty and staff concurred that social factors affected their patients’ health outcomes [35]. The higher levels of agreement in these studies likely result from samples that included respondents working in a wider variety of clinical positions.

While limited research exists on provider perceptions related to SDOH, studies show that most providers agree that social factors play an important role in their patients’ health [25, 28, 35]. Providers have been found to be generally supportive of attempts to address SDOH in the clinical setting [13, 25, 28, 36], yet studies show that most providers do not consistently ask patients about their social needs or review information on social needs when it is available in the patient chart [25]. While a proportion of providers (> 30% in our study) may not recognize the importance of SDOH to health outcomes, our results suggest that the most likely explanation for this gap between perception and practice is that most providers feel they lack the resources and capacity to adequately address SDOH [13, 25, 28, 36].

Findings from this study showed that most responses differed significantly by gender and specialty. In all but two survey items, female providers felt more strongly about the importance of SDOH and the value of SDOH screening than did male providers. These findings reflect that female physicians generally tend to be more patient centered, and engage more with their patients in psychosocial counseling, psychosocial questioning, and emotionally focused talk [37]. In addition, patients tend to communicate more to female physicians and disclose more psychosocial information [38].

Regarding differences between specialties, providers practicing general medicine, internal medicine, pediatrics, and psychiatry were more likely to view SDOH as important to patient health outcomes than those working in surgical specialties and rehabilitation medicine. Similarly, Palacio et al., found that primary care providers were more likely than specialists to agree that the benefits of collecting SDOH outweigh the risks, and that female faculty were more likely than male faculty to agree that SDOH collection would allow for the creation of special programs for at risk populations [28]. In a study of 602 pediatricians, Garg et al. found that females, minorities, generalists, and those practicing in rural areas were more likely to conduct routine screening [39].

These findings correlate with Lake et al.’s conclusions regarding the readiness of physicians and nurses to act on SDOH [40]. Despite the expressed comfort and confidence on SDOH, the willingness and preparedness to act is a complex interaction between personal attributes (skills, training, knowledge, values, attitudes), contextual factors (organizational and departmental culture, priorities, values, ideas, performance indicators), and situational factors (clinical encounters) [40]. The personal preparedness to tackle SDOH issues at the practice level may be explicitly or implicitly influenced by the priorities set by each specialty, which, may have been shaped by organizational and system readiness [41]. For instance, depending on their specialties, physicians and nurses may consider it an inherent responsibility to assess for and address SDOH as part of the treatment process, while others may perceive it to be outside of their professional role and responsibility or as peripheral to their department’s objectives.

Recently, Nausherwan Khan, et al. reported the results of a cross-sectional study exploring similar questions among providers within a major healthcare system in a single city from 2016 to 2017 [42]. Their participants reported little to no knowledge about SDOH. In contrast, respondents from our study reported they were familiar with the concepts of SDOH. There are several potential explanations for this discrepancy. Our sample was not limited to a single geographic area and may have captured a broader swath of experiences and perceptions. Additionally, a history effect may be at play based on when the different surveys were completed. It is likely that our survey respondents were more aware of SDOH due to a recent increased emphasis within the medical field but also because COVID-19 made SDOH more salient. This survey was conducted during the COVID-19 pandemic, which disproportionately affected historically marginalized groups [43, 44], further reinforcing existing health inequities and gaps in the spectrum of clinical care. These health disparities have been widely publicized in both scientific and popular media, bringing the discussion of SDOH to the forefront.

Screening patients for social concerns, followed by referral to community services and resources has been the major approach to integrating SDOH within the clinical spectrum of care. However, barriers exist to addressing SDOH more fully in the healthcare sector. Despite a consensus on the importance of SDOH in their respective practices and on the perceived influence of SDOH on their patients’ health outcomes, only 33.9% of our respondents reported highly prioritizing addressing SDOH in their own healthcare setting. Most respondents desired additional resources and/or programs to address patients’ unmet social needs. This aligns with the literature, as availability of medical resources and an appropriately trained healthcare workforce are considered important elements of a comprehensive approach to the SDOH in a clinical care setting [13, 15]. The degree of readiness to act on SDOH reported in this study may have been influenced by the increased demand on clinics and hospitals because of COVID-19. Adequately screening and addressing patients’ social factors, on top of pandemic-related stresses, can be time-consuming for providers who are already overburdened with the influx of COVID-19 patients [13].

The literature suggests that additional barriers to SDOH screening in the healthcare setting include the lack of standardized questionnaires and guidelines; failure to meet patients’ raised expectations [12, 20, 21]; patient discomfort in answering questions about non-medical needs; patient disinterest in receiving assistance on unmet social needs [24]; and the challenge of billing for and compensating healthcare providers for time spent on non-clinical services [13]. Some scholars question the ethical appropriateness of screening when clinics and hospitals have limited or no capacity to ensure linkage to appropriate services [20, 23]. Moreover, additional evidence is needed to recommend universal SDOH screening [21] since results are mixed regarding the effects of SDOH interventions on health outcomes, cost measures, and service utilization [36, 45, 46]. Furthermore, even though most providers in our study acknowledged the importance of SDOH, the proportion who did not was still sizeable at over 30%. This suggests that lack of buy-in from providers is another barrier to enhancing SDOH-focused practices in clinical settings, and it highlights the need for more comprehensive medical education acknowledging the importance of addressing social needs.

Despite the barriers to screening described above, in recent years, the medical field has begun to shift toward a view that recognizes the importance of SDOH within the healthcare context. This shift is being motivated by the well-established link between social factors and health as well as the growth in value-based reimbursement for healthcare [47]. SDOH questions were beta-tested in the Medical College Admission Test from 2013 to 2014 and were officially added in 2015. A 2017 survey of 32 medical schools that are part of the American Medical Association’s (AMA) Accelerating Change in Medical Education Consortium showed that while 74% of responding schools prioritized SDOH as high or extremely high, SDOH was not given the same attention as biomedical content [48]. Barriers to prioritization of SDOH within medical education included (in order of priority): (1) SDOH perceived as outside physician responsibility, (2) limited space within the curriculum, (3) faculty have limited training and experience with SDOH, and (4) the United States Medical Licensing Examination (USMLE) certifying examinations do not yet include questions about SDOH [48]. Such barriers will continue to have a direct downstream effect on the perceptions and training of medical providers and limit the likelihood providers will engage to understand and address SDOH with their patients.

Additional initiatives to better incorporate SDOH within the medical field vary widely. The American Medical Association is leading out on an initiative with United Healthcare to standardize SDOH data collection by creating 23 new IDC-10 codes (used to record diagnoses, symptoms, and procedures) specific to SDOH [49]. The AMA also supports efforts such as encouraging vendors of electronic medical records to incorporate SDOH fields and templates; developing standardized clinical exams to evaluate students’ skills around SDOH and cultural competence; and further integrating SDOH in medical education, including providing training for medical education faculty [49]. Resources specific to addressing SDOH within healthcare are also becoming more widely available, such as the Health Equity Resource Center by the National Committee for Quality Assurance (NCQA) [50].

The complex nature of both health and disease calls not only for a systems perspective in addressing SDOH within a healthcare setting, but also for cross-sectoral alignment and multisectoral efforts to achieve a common health agenda. Communities must have a system in place where community-based organizations and insurers can coordinate referrals, funding, and data [51]. To address these needs, several coordinated care platforms have been developed (e.g., Unite Us, Signify Community, NowPow, etc.) that provide a digital intermediary interface between healthcare organizations and social service providers [47]. These systems allow healthcare providers to send and receive referrals as well as track individual and community- level health outcomes. Landers et al. highlighted an approach to cross-sectoral alignment that was created by the Robert Wood Johnson Foundation (RWJF) in partnership with and coordinated by the Georgia Health Policy [41]. The aim is to develop effective strategies for aligning public health, health care, and social services in tackling individual and community needs, particularly in times of crises like the COVID-19 pandemic when demand on staff and the need for resources are high despite finite resources. Aligning efforts across multiple sectors requires meeting four elements: (1) defining a “shared purpose” and priorities that are informed by and shared by the community; (2) “shared data” as a foundation for coordinated efforts; (3) “long-term financing” with accountability; and (4) “robust governance structures” [41].

Efforts to align health systems in addressing SDOH also include establishing linking models of care between the community and clinical care as in the Accountable Communities of Health Model (ACH). As independent regional organizations, ACHs serve as the coordinating and connecting structures between communities and the healthcare delivery system to promote “whole-person health” by tackling SDOH inequities [15, 41, 52]. The ACH work with healthcare providers, local health units, and community-based organizations on specific public health, healthcare, and social needs-related problems through cross-sectoral discussions, workforce development, planning, value-based purchasing, integrating the care for physical and behavioral health, including investing in electronic health records [52].

This study provides valuable new insights into what healthcare providers from a variety of settings across the US believe about how SDOH influence their patients’ health. Findings, however should be interpreted considering the following limitations. First, our overall response rate was relatively low, and our sampling approach leaves open the possibility of sampling bias. While the sample was larger and more diverse than previous studies on this topic, the findings are not necessarily generalizable since participants self-selected to be part of the Reaction Data database and agreed to take the survey. Thus, the sample was not nationally representative of any demographic variable of interest. Second, while the study initially sought participants from a variety of healthcare backgrounds, nearly all respondents ended up being physicians, which is a limitation to these findings. In addition, there are other considerations (e.g., clinic resources, patient population, etc.) that are not the focus of this paper but that are relevant to how SDOH may or may not be integrated into clinical practice. Future research addressing these factors would be valuable. Third, while the various SDOH have been grouped together in a common framework that has proved useful for conceptual purposes, it is important to remember that, in practice, each determinant has unique characteristics with distinct effects on health outcomes. Some SDOH are more easily addressed by the healthcare sector, yet others may be much more difficult for healthcare providers to tackle. Future studies would be strengthened by questions that require providers to distinguish between different types of SDOH and report their perceptions and behaviors towards specific factors rather than SDOH as a whole.

## Conclusions

Most healthcare providers understand the importance of SDOH, and many are screening their own patients to identify and try to address unmet social needs. Female providers and those in certain specialties tend to prioritize SDOH more than other providers. It is important to improve the SDOH-related training that providers receive during their medical education and to further develop payment and delivery models that allow healthcare providers to take SDOH into account when caring for patients. However, poor SDOH stem from systemic problems that require systems-level solutions that go far beyond the healthcare sector and what clinicians can do for their own patients. Thus, efforts to address these problems require multisectoral approaches with significant coordination between healthcare organizations and community-based organizations that focus explicitly on SDOH. Healthcare providers with a clear understanding of SDOH can improve health outcomes by helping create these types of collaborations and by advocating for policy and systems changes that tackle the root causes of social problems.

### Electronic supplementary material

Below is the link to the electronic supplementary material.

Supplementary Material 1: Survey Instrument: Perceptions on the Social Determinants of Health. Description of data: File includes complete survey instrument as seen by respondents

## Data Availability

The datasets analyzed during the current study are available from the corresponding author on reasonable request.

## Abbreviations

AAFP: American Academy of Family Physicians
AAP: American Academy of Pediatrics
ACA: Patient Protection and Affordable Care Act
ACH: Accountable Communities of Health
ACO: Accountable Care Organization
AMA: American Medical Association
ASA: Agreed or Strongly Agreed
CMS: Center for Medicare and Medicaid Services
NACHC: National Association of Community Health Centers
NAM: National Academy of Medicine
NCQA: National Committee for Quality Assurance
RUCA: Rural-Urban Commuting Area
RWJF: Robert Wood Johnson Foundation
SDOH: Social Determinants of Health
US: United States
USMLE: United States Medical Licensing Examination
