# Supplementary material for: Do healthcare providers consider the social determinants of health? Results from a nationwide cross-sectional study in the United States
Source: BMC Health Serv Res. 2024 Mar 4;24:271. doi: 10.1186/s12913-024-10656-2 (PMC10910743; doi:10.1186/s12913-024-10656-2)
Supplement: Supplementary file 1 — Supplementary Material 1: Survey Instrument: Perceptions on the Social Determinants of Health. Description of data: File includes complete survey instrument as seen by respondents [file 12913_2024_10656_MOESM1_ESM.pdf]

Survey Instrument: Perceptions on the Social Determinants of Health

Q1. What is your gender?

a. Male
b. Female
c. Other (please specify)

Q2. What is your year of birth? ______

Q3. Which of the following best describes your race/ethnicity? Select all that apply.

a. Asian or Pacific Islander
b. Black or African American
c. Hispanic or Latino
d. Native American or Alaskan Native
e. White or Caucasian
f. Other

Q4. Please identify your level of agreement with the following statements:

*****SDOH = Social Determinants of Health.***
Note: Social determinants of health (SDOH) are defined as the conditions in the environments where people are born, live, learn, work, play, worship, and age; such as housing, transportation, education, income, food access, neighborhood safety, water and air quality, employment, etc (SDOH, 2020).

	Strongly Agree	Agree	Neither Agree or Disagree	Disagree	Strongly Disagree
a. I am confident in my understanding of SDOH.					
b. SDOH affects the health outcomes of **all** individuals in the US healthcare system					
c. SDOH affects the health outcomes among patients in **my** healthcare setting.					
d. SDOH are more important to overall health than the healthcare individuals receive.					
e. I highly prioritize addressing SDOH in my healthcare setting.					
f. Collecting information on SDOH will allow my healthcare setting to improve patient outcomes.					

g. Collecting information on SDOH would put additional burden on the providers in my healthcare setting.					
h. The benefits of collecting information on the SDOH outweigh the burden and risks.					
i. At the present time, my healthcare setting is set up to address the SDOH.					

Q5. Which individual or group do you feel should be responsible for identifying and addressing SDOH in your healthcare setting? Select all that apply.

a. Primary Care Provider (MD/DO, NP, RN, PA)
b. Population Health Team
c. Senior Healthcare Management
d. Administrative staff
e. SDOH are not the responsibility of those in healthcare settings
f. Other (please specify)

Q6. Please identify how often you do the following ***in your healthcare setting****:*

	All of the time	Some of the time	Occasionally	Rarely	Never
a. Think about the impact of SDOH on patients.					
b. Screen for SDOH.					
c. Address the SDOH of patients.					
d. Wish for additional resources/programs to address the SDOH of patients.					

Q7. In what ways does your practice currently help address a patient's SDOH, if any? (Select all that apply)

a. Screen for SDOH
b. Maintain up-to-date records of community-based resources
c. Refer patients to community-based resources
d. Engage patients about how to overcome their SDOH
e. Offer financial assistance programs
f. Offer transportation assistance programs
g. Offer housing assistance programs
h. Offer food assistance programs

i. Build cultural competence and proficiency
j. Offer health literacy or interpretation services
k. Other: ____________________________________

Q8. What systems, if any, do you have in place to ensure SDOH are addressed during patient visits? (Select all that apply)

a. Reviewing prompts in the electronic health record (EHR) system
b. Identifying SDOH as part of a patient's vital signs
c. Maintaining a registry of patients by categories of SDOH
d. Using flags or stickers on paper charts
e. Other: ____________________________________

Q9. What are the key barriers your practice experiences when addressing the SDOH? (Select all that apply)

a. Perceived lack of payment for SDOH
b. Staff expertise and capacity
c. Implicit bias and cultural proficiency
d. Lack of resources in patients' communities
e. Ensuring that patients know what to do and how to follow up with you
f. Engaging the healthcare team and building momentum
g. Lack of time
h. Other: _________________________________
